# Regulation of microRNA-200c in cancer stem cells

**DOI:** 10.18632/oncoscience.204

**Published:** 2015-08-20

**Authors:** Meng-Ju Wu, Mi Ran Kim, Chun-Ju Chang

**Affiliations:** Department of Basic Medical Sciences, College of Veterinary Medicine, Purdue University, Center for Cancer Research, Purdue University, West Lafayette, IN, USA

**Keywords:** breast cancer, cancer stem cell, microRNA

Stem cells are unspecialized cells that are not only able to differentiate into specialized cell types, but also to self-renew and infinitely reproduce themselves. Through impacting global gene expression, epigenetic regulators, such as microRNAs, play a crucial role in directing cellular programming to govern stem cell states (stemness vs. differentiation) [1-3]. Notably, during tumorigenesis, the generation of cancer stem cells (CSCs), a subset of the cancer cell population with acquired stemness properties associated with normal stem cells, allows the perpetual rise of the bulk of a tumor as the “seed” of the cancer. Earlier published studies have also provided strong evidence that an enlarged CSC population is associated with tumor aggressiveness and recurrence [2, 3]. Therefore, understanding the critical mechanism underlying regulation of cell stemness-differentiation homeostasis to sustain/exhaust the stem cell pool, which is likely to be shared between the normal mammary stem cells and the breast CSC population, holds the key to the development of effective therapeutic interventions to target CSCs and prevent cancer progression and recurrence.

The epithelial-mesenchymal transition (EMT) program is key to cancer progression and metastasis and has been linked to generation of CSCs [3]. Our previous work has shown that tumor suppressor p53 plays a role in regulating both EMT and EMT-associated breast CSCs through transcriptional activation of microRNAs involved in regulation of stemness, including microRNA-200c (miR-200c) [4]. Functional (wild-type) p53 transactivates miR-200c through direct binding to the miR-200c promoter, and loss of p53 in mammary epithelial cells leads to decreased expression of miR-200c and activates EMT program, accompanied by increased CSC-like population. Re-expressing miR-200c suppresses genes that mediate EMT and stemness properties and thereby reverts the mesenchymal and CSC-like phenotype caused by loss/mutation of p53 to a differentiated epithelial cell-like phenotype [4]. Notably, studies have shown that miR-200c is the most down-regulated miRNA in the normal and neoplastic stem cell populations compared with the non-stem cell populations [4, 5]. Furthermore, miR-200c plays an integral and active role in the maintenance of differentiated epithelia to antagonize tumorigenesis by directly targeting key proteins involved in activation of the EMT-CSC traits, such as ZEB1/2 and BMI1 [4, 5], and potentially more targets beyond. Therefore, determining the components and mechanisms underlying the regulation of miR-200c expression likely facilitates the development of strategies that can be used to pharmacologically manipulate miR-200c for effective cancer treatment.

It is recognized that obesity is an established risk factor for breast cancer incidence, progression, recurrence, and mortality, and that the dynamic interaction between tumor cells and the local microenvironment plays a crucial role in malignancy development. Interestingly, our recent study shows that in response to increased adiposity, elevated leptin, an adipokine produced by adipocytes, the most abundant cell type surrounding breast epithelia, activates STAT3-G9a to epigenetically silence a cohort of targeted genes and non-coding RNAs involved in regulation of cell differentiation and epithelial homeostasis, including miR-200c [6]. Down-regulation of miR-200c by activated leptin-STAT3-G9a signaling promotes the gain of EMT-CSC traits to expand a subset of highly tumorigenic breast CSCs enriched by the cell surface marker leptin receptor (OBRhi). In contrast, inhibition of STAT3 restores the expression of miR-200c, which in turn converts the CSC phenotype to a differentiated epithelial phenotype. Consistently, STAT3 inhibitor treatment significantly suppresses the CSC-like OBR^hi^ population and abrogates tumor progression of a diet-induced obesity rat model of breast cancer [6].

Together, these studies elucidate critical and sophisticated roles of intrinsic mutation (e.g. loss of p53) and extrinsic influence (e.g. increased adiposity) in directing EMT-MET (mesenchymal epithelial transition) and stemness-differentiation plasticity through regulation of miR-200c expression during tumorigenesis. The results further reveal novel therapeutic implications of re-activation of p53 or inhibition of STAT3 in restoration of miR-200c to eliminate the CSC pool and thereby prevents breast cancer progression/recurrence.
